# A microfluidic platform for continuous monitoring of dopamine homeostasis in dopaminergic cells

**DOI:** 10.1038/s41378-019-0049-2

**Published:** 2019-03-11

**Authors:** Yue Yu, Richard P. S. de Campos, Seolim Hong, Dimitar L. Krastev, Siddharth Sadanand, Yen Leung, Aaron R. Wheeler

**Affiliations:** 1 Institute for Biomaterials and Biomedical Engineering, University of Toronto, 164 College St, Toronto, ON M5s 3G9 Canada; 2 Donnelly Centre for Cellular and Biomolecular Research, 160 College St., Toronto, ON M5S 3E1 Canada; 3 Department of Chemistry, University of Toronto, 80 St George St., Toronto, ON M5S 3H6 Canada; 4 Department of Human Biology, University of Toronto, 300 Huron Street, Toronto, ON M5S 3J6 Canada

**Keywords:** Chemistry, Engineering

## Abstract

Homeostasis of dopamine, a classical neurotransmitter, is a key indicator of neuronal health. Dysfunction in the regulation of dopamine is implicated in a long list of neurological disorders, including addiction, depression, and neurodegeneration. The existing methods used to evaluate dopamine homeostasis in vitro are inconvenient and do not allow for continuous non-destructive measurement. In response to this challenge, we introduce an integrated microfluidic system that combines dopaminergic cell culture and differentiation with electroanalytical measurements of extracellular dopamine in real*-*time at any point during an assay. We used the system to examine the behavior of differentiated SH-SY5Y cells upon exposure to four dopamine transporter ant/agonists (cocaine, ketamine, epigallocatechin gallate, and amphetamine) and study their pharmacokinetics. The IC_50_ values of cocaine, ketamine, and epigallocatechin gallate were determined to be (average ± standard deviation) 3.7 ± 1.1 µM, 51.4 ± 17.9 µM, and 2.6 ± 0.8 µM, respectively. Furthermore, we used the new system to study amphetamine-mediated dopamine release to probe the related phenomena of dopamine transporter-mediated reverse-transport and dopamine release from vesicles. We propose that this platform, which is the first platform to simultaneously evaluate uptake and release, could be useful to screen for drugs and other agents that target dopaminergic neurons and the function of the dopamine transporter. More broadly, this platform should be adaptable for any application that could benefit from high-temporal resolution electroanalysis combined with multi-day cell culture using small numbers of cells.

## Digital microfluidics: detecting dopamine

A microfluidic-based setup permits real-time in-vitro assessment of cellular dopamine uptake. Regulation of dopamine in the body is crucial to the health of the nervous system; ineffective regulation can result in damage to neurons and a range of neurological conditions, including addiction and Parkinson’s Disease. A key process in the regulation of dopamine is its release/uptake from cells. Now, a team led by Aaron Wheeler from the University of Toronto report a digital microfluidic device that integrates in-vitro cell culture with electrochemical analysis, in order to detect dopamine uptake and release in real-time during an assay. This new system might also be useful for screening libraries of reagents for the identification of new drugs.

## Introduction

The maintenance of dopamine (DA) homeostasis in the nervous system is critically important as DA overexposure can lead to excitotoxicity and neuron death^1^. The complex mechanisms that maintain DA homeostasis include a delicate balance between intracellular processes, such as DA synthesis, vesicle packing and degradation, and inter/extra cellular processes, such as the release and uptake of DA^2^. This paper focuses primarily on the latter. The membrane-bound dopamine transporter (DAT), which actively uptakes extracellular dopamine from the extracellular milieu, is a key player in inter/extracellular DA homeostasis^3^. Thus, DAT dysfunction and the loss of DA homeostasis (caused by DAT binding to misformed parkin^4^ or α-synuclein^5^ or DAT-modulation by agonists such as cocaine^6^) are associated with debilitating disorders, such as addiction^7^ and Parkinson’s disease^8^.

Our understanding of DA homeostasis is based on evaluations of both individual neurons^9–11^ and bulk cell samples^12^ (i.e., tissue slices or large numbers of cultured cells). Single-cell studies, which rely on finely tuned systems combining high-resolution imaging, sub-micron XYZ-manipulators, and pulled-pipette-encased DA-sensors, are useful for resolving individual exocytotic events^10,11^, permitting the assessment of the fundamental unit of neurotransmission. Bulk cell studies are useful because of the ability to apply rigorous experimental controls, enabling the determination and comparison of the kinetics of ant/agonists on DAT and other DA homeostasis-regulators. Bulk cell studies can be sub-divided into studies relying on direct methods^12^ (in which labeled DA is quantified in cell lysate after uptake) or indirect methods^13^ (in which extracellular DA is quantified before and after uptake). The readout of the former is often a scintillation counter, while the latter typically involves immunoassays implemented in a well plate. These techniques are tedious, incapable of real-time analysis and subject to experimental bias because of the susceptibility of DA to auto-oxidation^14^. Thus, researchers who study DA regulation are in need of new tools that allow for robust, real-time measurements of DA uptake in a format that is compatible with repeatable controlled studies.

In response to the challenge described above, we developed an automated digital microfluidic (DMF) platform that integrates in vitro cell culture^15,16^ with electrochemical analysis^17,18^. We recently described an initial solution to this problem^19^, i.e., a microfluidic device that includes (1) a cell-culture module designed for multi-day dopaminergic neuron culture and (2) a quantitative amperometric DA sensor (“e-sensor”) module. A key limitation to this previous method^19^ is its modular nature, i.e., droplets of culture medium are shuttled between the two modules, rendering continuous measurements impossible. Here, we introduce a substantially improved system that allows for multi-day cell culture and differentiation with real-time continuous measurements of DA uptake. Critically, the new system allows for the measurement of the net DA uptake and release (as a proxy for DA homeostasis) in a time-resolved manner. We propose that future generations of this system could be useful for screening libraries of reagents to identify potential new drugs and/or therapeutic targets for DA (or other electroactive neurotransmitter) dysregulation disorders.

## Results and discussion

### Microfluidic system for cell culture and continuous DA uptake measurements

The goal for this work was to develop a method that allows for continuous, time-resolved measurements of DA homeostasis in dopaminergic cells. Numerous microchannel-based microfluidic platforms have been used to evaluate neurotransmitter release from neuronal cells^20,21^, but these platforms have not been applied to this problem; moreover, we are unaware of any system that is capable of evaluating both neurotransmitter uptake and release. The closest system was our previous method^19^, which had separate modules for (a) cell culture and (b) DA electroanalysis. Here, we sought to combine (a) and (b) into a single, integrated unit known as a “cell-culture/electroanalytical sensor” (or “c-e-sensor”) that allows for the continuous monitoring of DA homeostasis.

**Fig. 1:**
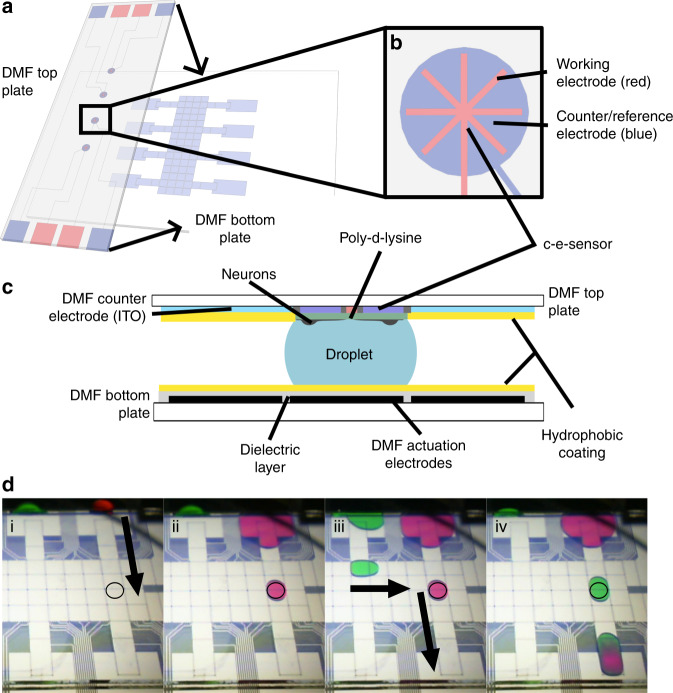
DMF device for dopaminergic cell culture with integrated c-e-sensor for the continuous measurement of DA uptake. **a** Isometric-view schematic of DMF top plate over a DMF bottom plate (black arrows indicate how the two plates are assembled). The conductive ITO of the top plate is patterned into four c-e-sensors, each bearing a working (WE, red) and counter/pseudoreference (CE/RE, blue) electrode. The remainder of the top plate contains one large, irregular DMF counter-electrode. **b** Top-view cartoon of a c-e-sensor denoting the WE and CE/RE. **c** Side-view schematic of an assembled DMF device bearing a droplet positioned on the e-sensor. Notably, the entire c-e-sensor (including both the WE and CE/RE) is coated with a layer of poly-d-lysine (green) to promote neuron attachment. **d** Frames from a movie illustrating basic droplet movement and passive dispensing on DMF. The open black circle indicates the position of the c-e-sensor. i–ii A droplet of liquid 1 (red food coloring dye dissolved in PBS) is dispensed from a reservoir electrode on the right and actuated across the c-e-sensor, passively dispensing a VM onto it. iii–iv A droplet of liquid 2 (green food coloring dye dissolved in PBS) is dispensed from a second reservoir and driven across the c-e-sensor to displace liquid 1, filling the VM with liquid 2

The system introduced here is shown in Fig. 1. Figure 1a illustrates the assembly of a DMF top plate bearing four e-sensors with a standard DMF bottom plate. The DMF top plate was patterned into the following five distinct regions: (1) a large irregularly shaped indium tin-oxide (ITO) region coated with hydrophobic Teflon AF acting as the DMF counter electrode and (2) four circular 2 mm diameter hydrophilic Teflon AF liftoff spots bearing the c-e-sensors. Figure 1b shows a magnified illustration of a single c-e-sensor, i.e., a two-electrode system comprising an ITO working electrode (WE, red) and an ITO counter/pseudoreference electrode (CE/RE, blue). Externally, this electroanalytical cell is linked to a home-built, open-source potentiostat^22^ that interfaces directly with a home-built, open-source DMF automation system^23^. In our previous work^19^, we established two criteria required for enhancing the WE stability and sensitivity for DA measurements. First, to enhance stability and minimize the voltage drift, the area of the WE must be at least 10×smaller than the area of the CE/RE. Second, to enhance sensitivity, the WE-CE/RE interface should be long, which is a strategy that has been widely applied in micro-band electrode systems^24^. The c-e-sensor used here was designed based on our previous design^19^, and the WE and CE/RE areas were 1.19 and 11.9 mm^2^, respectively. Additionally, a star-shaped WE was used, and the total perimeter (defined as the length of the interface between WE and CE/RE) was 11.85 mm.

Figure 1c provides a side-view schematic of an assembled DMF device with a droplet on a single c-e-sensor. In an assembled DMF device, the droplets are manipulated across an open surface via electrostatic forces generated between the DMF actuation electrodes (black) on the bottom plate and the DMF counter electrode (light blue) on the top plate. Figure 1d illustrates how the device is used in practice. As previously described^25^, when a droplet is driven across a hydrophilic site on a surface that is otherwise hydrophobic, a sub-droplet (known as a “virtual microwell” or VM) is spontaneously formed on the site via a process known as passive dispensing. In the example shown in Fig. 1d, a 1.2 µL droplet is driven across a c-e-sensor, forming a 470 µL VM; subsequently, the contents of the VM are exchanged. It has been previously shown^15^ that 2–3 passive dispensing steps are sufficient to completely remove any traces of analytes in the original solution from the VM. In practice, SH-SY5Y cells were seeded, cultured, and differentiated on c-e-sensors as described in the supplementary information, and then, DMF and passive dispensing were used to automate all experiments described below.

The new system described above (Fig. 1) integrates cell culture and electroanalysis onto the same surface to enable continuous measurements in cultured cells. The following are two critical questions regarding this type of system: does the integration adversely affect cell health and differentiation? Does the integration adversely affect the electroanalytical measurement?

**Fig. 2:**
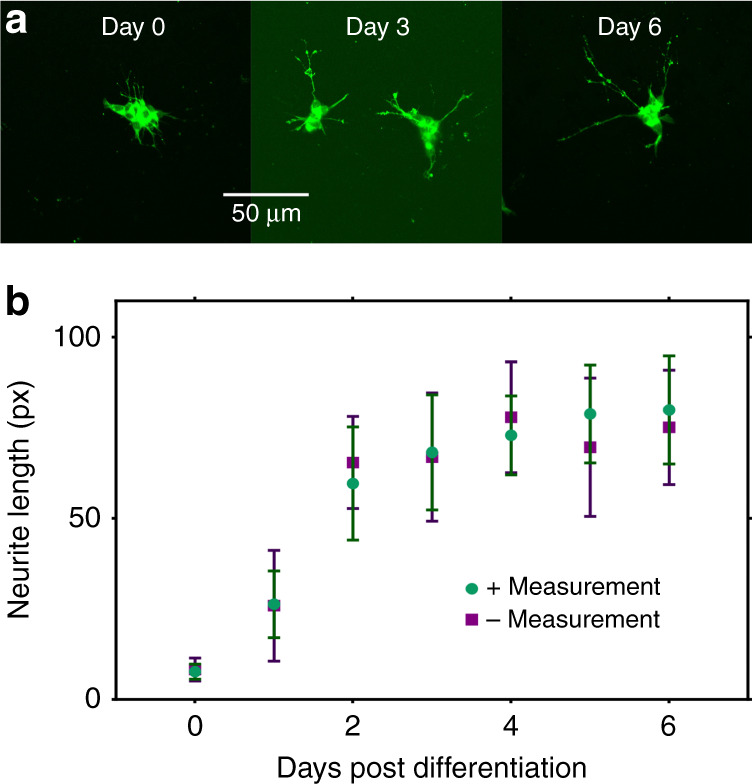
Effect of electroanalysis measurements on SH-SY5Y health and differentiation in cells cultured on c-e-sensors. **a** Representative fluorescence images on days 0, 3 and 6 of differentiation in SH-SY5Y cells labeled with βIII-tubulin (green) cultured on an electroanalytical sensor. Scale bar is 50 µm. **b** Plot of neurite lengths in SH-SY5Y cells grown and differentiated on c-e-sensors with (green circles) and without (purple squares) exposure to daily electroanalytical measurements (5 differential pulse voltammograms, scanning from 0 to 0.9 V at 50 mV/s). Error bars represent ± 1 S.D. *N* *=* 150 cells were selected randomly from 10 c-e-sensors on 3 top plates

We probed the first question via a series of experiments by subjecting SH-SY5Y cells to daily measurements with differential pulse voltammetry (DPV; parameters chosen to mimic those used in DA uptake experiments as described below) at various stages of differentiation. As shown in Fig. 2a, SH-SY5Y cells grown on c-e-sensors exhibit the predicted phenotype. Day-0 cells display faint, short, and thin processes consistent with an immature neuron phenotype, while day−3 and −6 cells exhibit a phenotype that is more consistent with that of mature neurons (i.e., thicker and longer neurites expressing βIII-tubulin). To assess the effect of electroanalysis, cells at varying stages of differentiation were interrogated daily with DPV; then, 150 randomly chosen neurons across 10 e-sensors from 3 top plates were evaluated by microscopy. The average neurite lengths in these cells were compared to those in neurons grown on c-e-sensors that were not ‘measured.’ As shown in Fig. 2b, the neurite lengths of the differentiating SH-SY5Y cells that were subjected to daily DPV measurements did not significantly differ from their un-measured counterparts (*p* = 0.9675). While this is not a comprehensive measure of cell health and fitness, the neurite length is a sensitive marker of the neuron phenotype^26^, and these results suggest (at least) that daily electrochemical measurements do not impede SH-SY5Y differentiation into a neuron-like phenotype. Notably, this outcome is not surprising as the voltage used and the current generated by the DPV measurements described here are low, with the maximum voltage never exceeding 0.9 V and current consistently below 400 nA. For comparison, most electrophysiology experiments are performed in the range of 2–5 V and/or above 10 µA^27^.

**Fig. 3:**
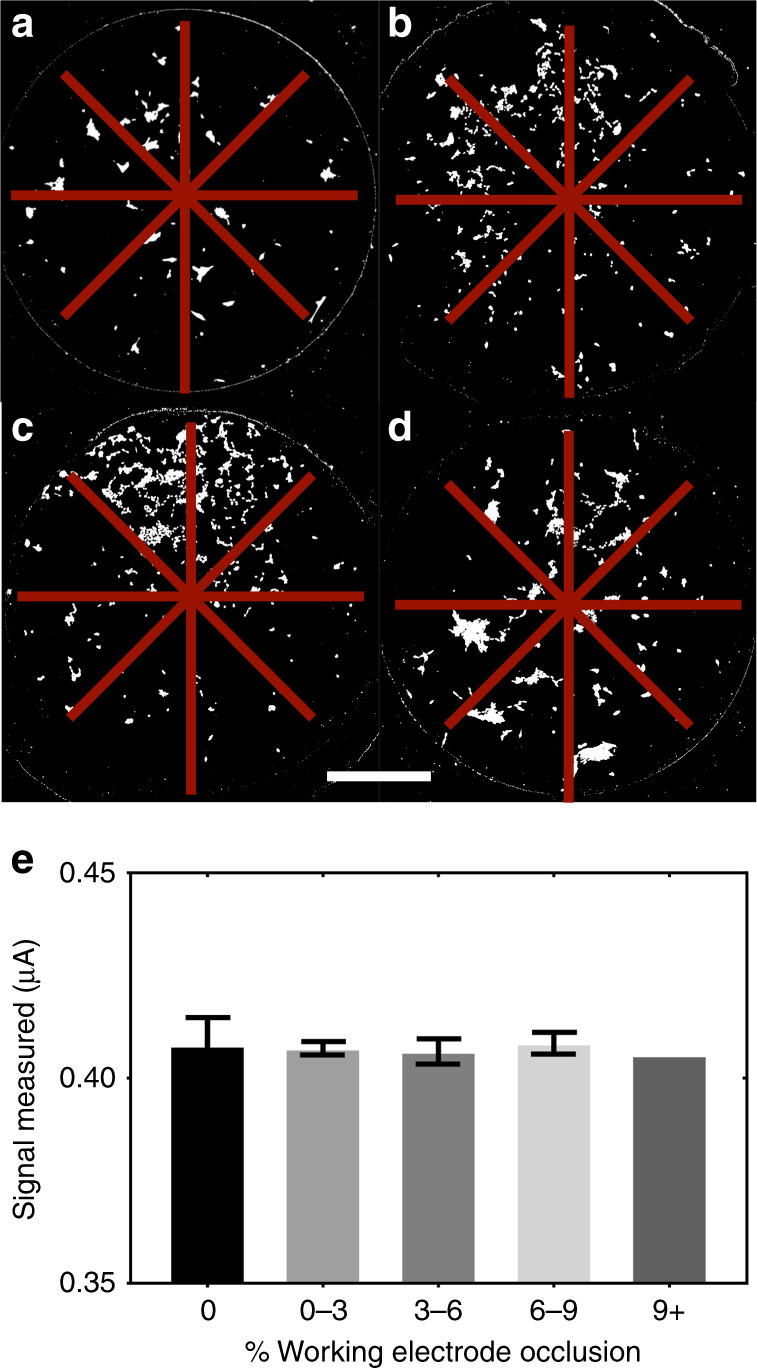
Effect of SH-SY5Y cells on the electroanalytical performance of the c-e-sensor. Representative confocal fluorescence images of SH-SY5Y cells seeded at (**a**) 200, (**b**) 1000, (**c**) 2500, and (**d**) 5000 cells/c-e-sensor. The cells were differentiated for 6 days and then fixed and stained for βIII-tubulin (white). The WEs are indicated in dark red, and the scale bar is 500 µm. **e** Bar graph comparing the DPV signal measured from a droplet of 1 µM DA on c-e-sensors seeded with varying numbers of cells binned according to the % WE occlusion. The left-most bar (black) represents bare electrodes not seeded with cells; the bars on the right (shades of grey) represent bins generated from cells seeded on c-e-sensors in varying densities (as shown in panels **a**–**d**). Error bars represent ± 1 S.D. *N* *=* 6 (0%), *N* *=* 5 (0–3%), *N* *=* 7 (3–6%), and *N* *=* 7 (6–9%). In the 9 + % bin, *N* *=* 1

After confirming that the protocol of repeated daily DPV measurements did not result in an obvious impact on SH-SY5Y differentiation, we focused our attention on the potential effects of cells on the electrochemical DA measurements. It is well known that voltammetric analysis sensitivity depends on the total active WE surface area exposed to the sample^24^. In our previous work using separate modules for cell culture and electroanalysis, the electrodes were completely unobstructed (i.e., the cells were cultured elsewhere). However, in the current system, the WE is (by necessity) partially covered by growing cells, which could reduce the active WE surface area. To determine the effect of cells covering the WE on the signal measured by the c-e-sensor, we loaded SH-SY5Y cells at different densities and measured the DPV signal observed from a 1-μM solution of DA at 20 Hz (mimicking an uptake experiment as described below). Confocal fluorescence images of the cells were also obtained (Fig. 3a-d) and used to calculate the “fractional coverage” value, which represented the surface area of the WE covered by the SH-SY5Y cells. These images were binned according to the fractional coverage in sets of 0% (control with no cells), 0–3, 3–6, 6–9, and 9+%. As shown, there is no significant difference between the no-cell control and all the occlusion conditions (one-way ANOVA, *p* = 0.86). This finding suggests that in cases with up to 9% occlusion of the WE with cells, the error in the signal measured by the c-e-sensor is not caused by cell-occlusion-related changes in the WE area. Since all DA-uptake experiments described below had a WE occlusion ≤ 4%, we are confident that the measured signals were not observably affected by the presence of cells on the c-e-sensors.

We propose that the observed (non-)effect of the cells adhered to the electroanalysis electrodes on the DPV measurements is not surprising. At the frequencies used here (20 Hz), the conductivity of the medium dominates the circuit relative to the small amount of occlusion caused by the sparsely populated cells. In fact, in similar systems, the presence of cells grown on electroanalytical electrodes has been observed to effect measurements only when the frequency of the applied voltage is high^28^ (>1 kHz). These types of high-frequency measurements are useful for impedimetric analyses of the cell shape, size, and viability^29^ (and have even been used in DMF systems^30^) but are quite different from the low-frequency DPV analysis employed here.

### DA uptake in SH-SY5Y cells

**Fig. 4:**
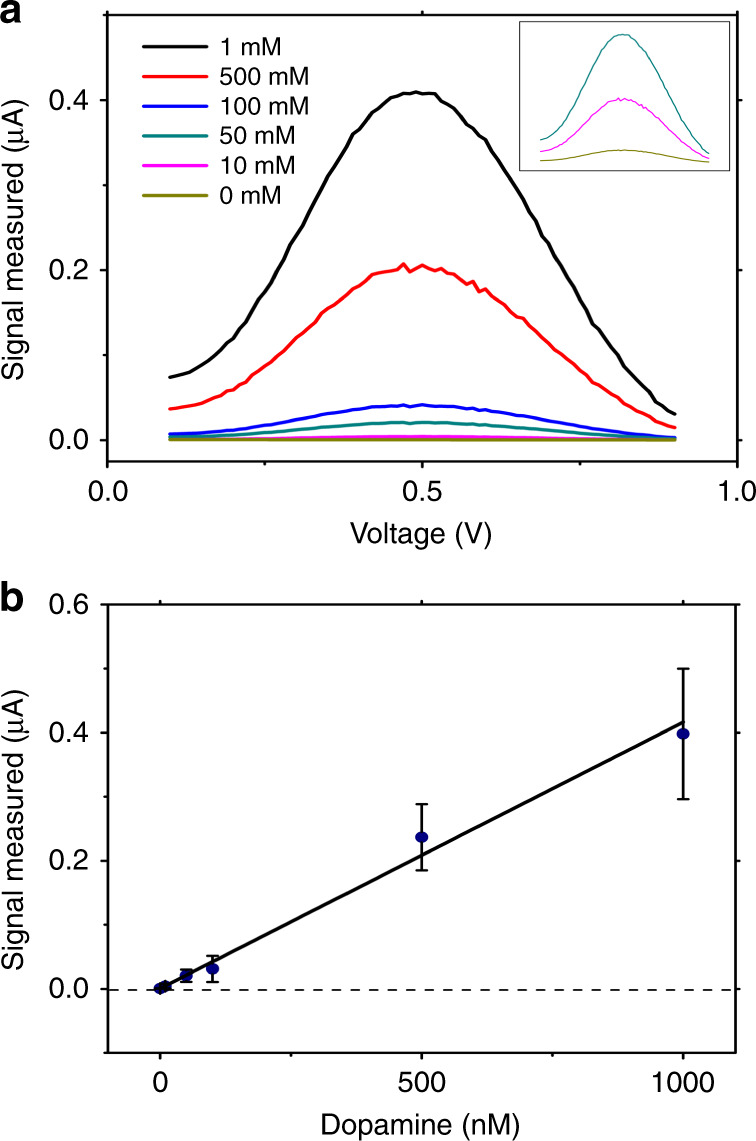
Characterization of DPV measurement of extracellular DA concentration on the c-e-sensor. **a** Representative differential pulse voltammograms of DA at 1 µM (black), 500 nM (red), 100 nM (blue), 50 nM (cyan), 10 nM (pink), and 0 nM (1 × PBS solution, brown). The inset shows a magnified view of the lowest three concentrations (50, 10, and 0 nM). **b** Calibration curve generated by measuring the currents at 0.5 V with standards ranging from 0 nM to 1 µM DA. Error bars represent ± 1 S.D. *N* *=* 3 per condition. The limit of detection (defined as the concentration corresponding to the blank signal plus 3 × the standard deviation of the blank) was determined to be 11.6 nM

After demonstrating that growing cells on the surface of the c-e-sensor electrodes do not negatively affect the electroanalytical measurements and vice versa, we evaluated the utility of the system for DA uptake experiments. In our previous system^19^, we used cyclic voltammetry to quantify DA. While cyclic voltammetry is an effective method for this purpose, it is susceptible to voltage drift and is influenced by the charging current^24^. Thus, in the system reported here, we chose to explore the use of differential pulse voltammetry (DPV), which corrects for both susceptibilities^31^. Figure 4a shows representative differential pulse voltammograms of DA at different concentrations in phosphate buffered saline (PBS) recorded using the system with the singular peak at 0.5 V. This peak potential differs from that in other reported electroanalytical studies of DA primarily because of the difference in the electrode setup and material (i.e., a 2-electrode ITO vs ITO setup here, 3-electrode Carbon fiber vs Ag/AgCl in the other studies^31^). As shown in Fig. 4b, the DPV signal recorded at 0.5 V is linear with respect to the increasing DA concentration (*y* = 0.00042 × + 0.00113, *R*^2^ = 0.9934), and the limit of detection is 11.6 nM.

**Fig. 5:**
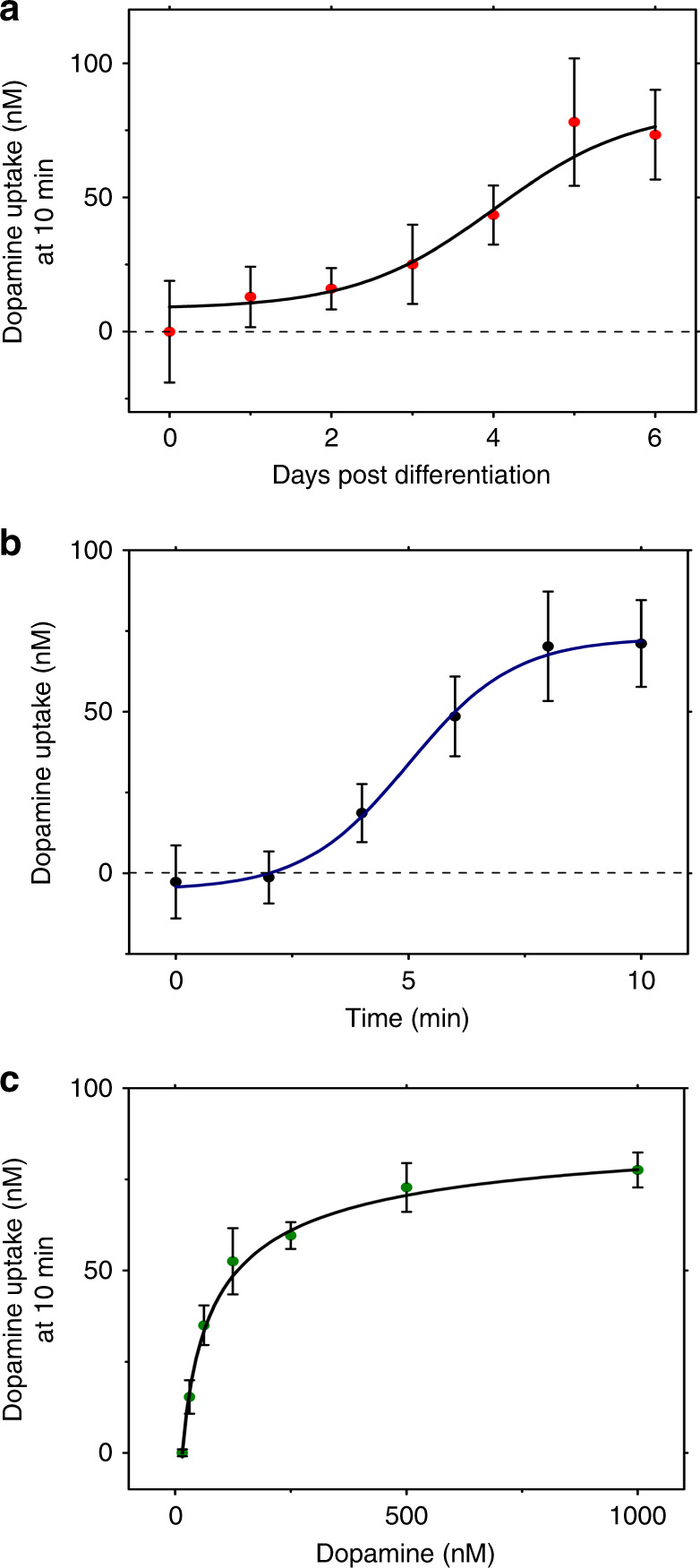
Application of DMF and c-e-sensors for electroanalytical measurements of DA uptake. **a** DA uptake (red circles) with sigmoid fit (black curve) recorded every 24 h from SH-SY5Y cells cultured on c-e-sensors during differentiation. Each measurement was performed after a 10-min incubation with 1.0 µM DA. Error bars represent ± 1 S.D. *N* *=* 5 cell-laden c-e-sensors. **b** Continuous measurements of DA uptake (black circles) with sigmoid fit (blue curve) in day-6 differentiated SH-SY5Y neurons incubated with 1.0 µM DA. Error bars represent ± 1 S.D. *N* *=* 12 cell-laden c-e-sensors. **c** DA uptake (green circles) with Hill-equation fit (black curve) in day-6 differentiated SH-SY5Y neurons after incubation with 0.015, 0.03125, 0.0625, 0.125, 0.25, 0.5, or 1.0 µM DA for 10 min. Error bars represent ± 1 S.D. *N* *=* 3 cell-laden c-e-sensors. Parameters extracted from the curve include *V*_max_ = 78.6 ± 0.6 nM, *K*_h_ = 86.0 ± 19.6 nM, and *n* = 1.6 ± 0.6

As a first test of the new system, we applied it to evaluate DA uptake over the course of the 6-day SH-SY5Y differentiation process. In these experiments, the cells were periodically removed from the incubator and exposed to a droplet of 1 μM DA, and uptake was measured after 10 min. As shown in Fig. 5a, immature (day-0) SH-SY5Y cells do not significantly interact with extracellular DA, but these cells progressively become sensitive to DA on days 2–4, and a relatively stable amount of uptake is observed on days 5–6. This trend matches the results obtained using our previous method^19^ in which the concentration of measured extracellular DA is statistically unchanged before/after exposure to immature SH-SY5Y cells or other non-dopaminergic cells. Stated another way: reductions in extracellular DA are only observed after exposure to differentiated SH-SY5Y cells, which increases our confidence that this phenomenon is a result of DA uptake (and not auto-oxidation or adsorbance onto the device surfaces) and is likely a consequence of DAT activity in the cell membrane^12^. Day-6 differentiated cells were used in all experiments reported below.

Next, we focused on the most unique and important feature of the new method relative to previously reported methods^19^, i.e., the capacity to perform uptake measurements in situ with high time resolution. Figure 5b demonstrates the monitoring of DA uptake by differentiated SH-SY5Y cells immediately after dosing with a droplet of 1 μM DA. In these experiments, the measurements were obtained every 2 min (which was deemed sufficient for the observed kinetics), but because the cells and electroanalytical electrodes are co-located, the measurements can be obtained at any frequency dictated by the application (e.g., in the other experiments, as described below, the measurements were performed every 30 s). This method proved particularly useful for evaluating the effects of DAT inhibitors (see below).

Finally, we evaluated cells exposed to DA at different concentrations (after 10-min incubation) to examine the kinetics of DA uptake. Figure 5c shows a Hill curve of DA uptake with a maximal uptake rate *V*_max_ = 78.6 ± 0.6 nM and an initial concentration of DA required for half-maximal uptake *K*_h_ = 86.0 ± 19.6 nM. We chose a Hill-equation fit rather than the Michaelis-Menten fit (which has previously been used for DA uptake^12^) because DA uptake does not follow traditional first order enzyme kinetics^32,33^. Specifically, DAT is known to form tetramers (dimers of dimers) on the cell membrane, suggesting that allosteric modulation occurs between each individual DAT subunit in the complex^32,33^. This notion is supported by the Hill-equation fit reported here (*R*^*2*^ = 0.9687), which has a cooperativity of *n* = 1.6 ± 0.6 (ave. ± S.D., *N* = 3).

The data shown in Fig. 5 is qualitatively consistent with previous reports with some differences in quantities. For example, the *V*_*max*_ was calculated to be equivalent to 201 ± 24 pmol DA uptake/mg of cellular protein (see supplementary information for details); this value is approximately 6 × higher than that previously reported in SH-SY5Y cells using alternate methods^12^. We attribute this discordance to differences in the experimental protocol, including (1) the use of indirect electrochemical determination in the media (here) instead of direct radiometric analysis of [^3^H]DA in the cell lysate^12^, (2) the rapid nature of the new method (which should limit the amount of DA oxidation) and (3) the integrated nature of the new method, which should limit the amount of DA loss to non-specific adsorption in multiple transfers among plates, tubes, and pipettes. Notably, a recent survey investigating DAT-mediated DA uptake in SH-SY5Y cells and other model systems^34^ suggests that there are wide variances reported for *V*_max_ and other parameters, underscoring the need for standardized tools that can be replicated in different settings. We propose that the methods described here, which were implemented using open-source instrumention^22,23^ (and are freely available to anyone who wishes to use these methods), represent a useful step toward this goal.

### Continuous DA uptake measurements with DAT ant/agonists

After validating the c-e-sensor for DA uptake measurements, we applied the new platform to pilot screen three drugs [cocaine, ketamine, and amphetamine (AMPH)] and a nutraceutical [epigallocatechin-3-gallate (EGCG), extracted from green tea] that have been reported to disrupt DA homeostasis. Three of the four agents (cocaine^35^, EGCG^36^, and ketamine^37^) are known DA uptake inhibitors, while AMPH^38^ is an agonist of DA secretion. The screening involved the co-incubation of the four compounds (at various concentrations) with DA on differentiated SH-SY5Y cells with continuous electroanalytical monitoring to evaluate the effects of each agent on DA uptake.

**Fig. 6:**
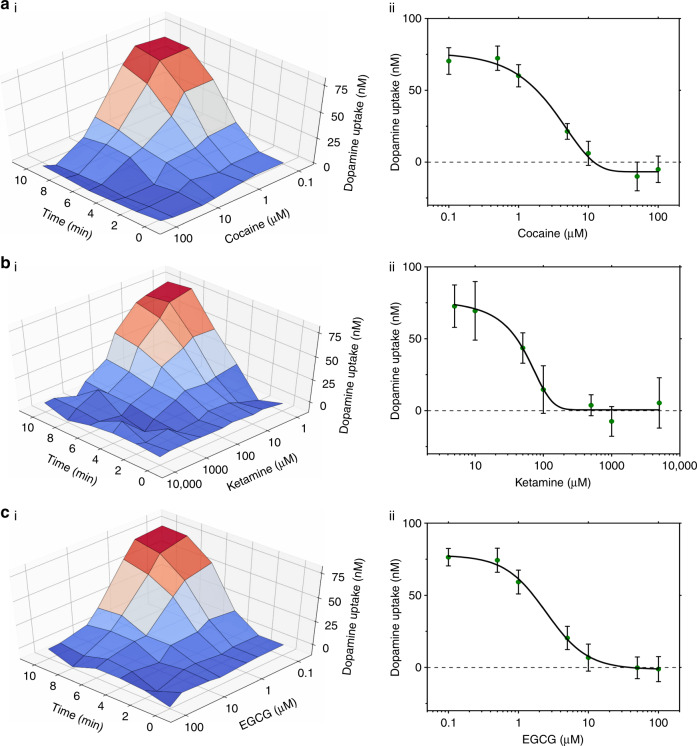
Application of DMF and c-e-sensors for the screening of the effects of DAT inhibitors on DA uptake. Day-6 differentiated SH-SY5Y neurons were incubated with droplets containing 1.0 μM DA plus **a** 0, 0.1, 0.5, 1.0, 5.0, 10, 50, or 100 µM cocaine, **b** 0, 1.0, 5.0, 10, 50, 100, 500, 1000, or 5000 µM ketamine, or **c** 0, 0.1, 0.5, 1.0, 5.0, 10, 50, 100 µM EGCG in 1 × PBS for 10 min, and DA uptake was measured every 2 min. (i) Three-dimensional surface-curves of extracellular DA measured relative to the inhibitor concentration and incubation time. A heat-map from red to blue indicates the conditions under which DA uptake is observed or inhibited. (ii) Dose-response slices (data in green circles with black-line sigmoid fits) based on the data shown in (i) collected after 10 min of incubation. Error bars represent ± 1 S.D. *N* *=* 12 (cocaine and EGCG) and 9 (ketamine) cell-laden c-e-sensors per condition

Cocaine is commonly used as a positive control in DAT-inhibition assays and has been widely studied because of its addictive nature^39^. Pharmacologically, cocaine acts as a competitive inhibitor that binds and locks DAT in a non-functioning isoform^35^. Figure 6ai shows aggregate data collected from time-course experiments using a range of cocaine concentrations, and Figure 6aii shows the dose-response curve over a 10-min incubation (*R*^2^ = 0.9903). As expected, at low concentrations of cocaine, SH-SY5Y neurons uptake DA (as indicated in Fig. 5), but at high concentrations, the uptake is completely inhibited. Based on these data, the IC_50_ was found to be 3.7 ± 1.1 µM (ave. ± S.D. for cells on 12 different c-e-sensors), which is consistent with the low micromolar IC_50_ values previously reported for cocaine^40^. Ketamine is commonly used to induce and maintain general anesthesia;^41^ similar to cocaine, ketamine is addictive at high dosages and has the ability to address treatment-resistant depression, implying that it also interacts with DA homeostasis^37,41^. Figure 6bi shows the aggregate response, and Figure 6bii shows the dose-response curve of ketamine (*R*^2^ = 0.9530) with IC_50_ = 51.4 ± 17.9 µM (ave. ± S.D. for cells on 9 different c-e-sensors). This higher IC_50_ value (relative to that of cocaine) is consistent with findings indicating that a high ketamine concentration (in the order of 100 µM) is needed to decrease DA uptake in primary brain slices^37^. EGCG is a non-pharmaceutical DAT inhibitor^36^ that has been studied as a potential source of the reputed health benefits of green tea^42^. While the mechanism by which EGCG inhibits DAT is unknown, one hypothesis is that EGCG molecules form a complex with DAT, causing the complex to internalize, where it can no longer uptake DA^36^. Figure 6ci shows the aggregate response, and Figure 6cii shows the dose-response curve of EGCG (*R*^2^ = 0.9959) with IC_50_ = 2.6 ± 0.8 µM (ave. ± S.D. for cells on 12 different c-e-sensors). The IC_50_ of EGCG determined here is consistent with previous reports of the inhibition of DA uptake when incubating DAT-PC12 cells with EGCG concentrations ranging from 1–100 μM^36^.

**Fig. 7:**
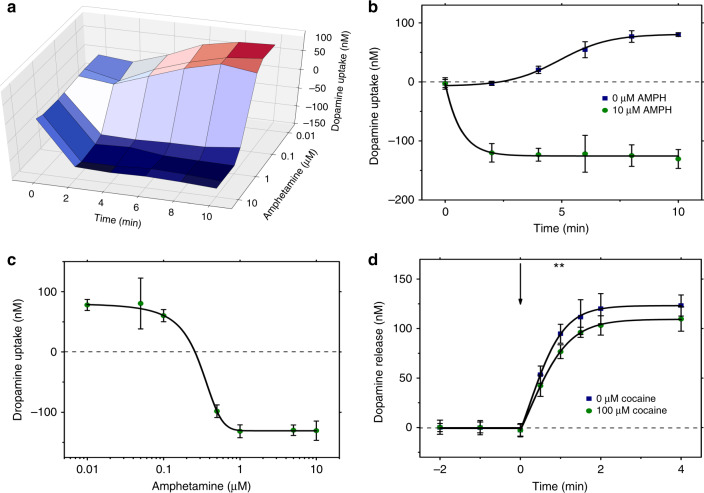
Application of DMF and c-e-sensors to screen for the effect of AMPH on DA homeostasis. **a** Three-dimensional (3D) surface-curve of extracellular DA measured relative to the AMPH concentration and incubation time of day-6 differentiated SH-SY5Y neurons incubated with a unit droplet containing 1.0 μM DA, plus 0, 0.1, 0.5, 1.0, 5.0, or 10 µM AMPH in 1 × PBS for 10 min. A heat-map from red to dark blue indicates the conditions under which DA uptake or release is observed. **b** Continuous measurements of DA uptake from the data shown in **a** in cells incubated with 0 µM (blue) or 10 µM (green) AMPH in 1 × PBS with sigmoid fits (black lines). **c** Dose-response slice (data in green circles with black-line sigmoid fits) from the data shown in **a** obtained after 10 min of incubation. Error bars in (**b**, **c**) represent ± 1 S.D. *N* *=* 12 cell-laden c-e-sensors per condition. **d** Comparison of day-6 differentiated SH-SY5Y neurons that have been pre-incubated for 10 min with (green circles) or without (blue squares) 100 µM cocaine with sigmoid fits (black lines); error bars represent ± 1 S.D. *N* *=* 9 cell-laden c-e-sensors per condition. After the pre-incubation, the cells were incubated with 1.0 μM DA + 10 µM AMPH in 1× PBS. The black arrow represents the time AMPH was introduced to the neurons. The double star represents a significant difference (*p* = 0.0024) between the extracellular DA concentrations between the two conditions measured after 1 min of incubation

After evaluating the DA uptake inhibitors, we focused our attention on evaluating the effects of AMPH, which is a known agonist of cellular DA release^38^. Notably, the ability to simultaneously monitor all aspects of DA homeostasis (including uptake and release) is a unique feature of this “indirect” label-free method that is not possible for the more common “direct” methods^12^ (which can only detect uptake as labeled analyte in the cell lysate). Figure 7a shows the aggregate data obtained from the time-course experiments using a range of concentrations of AMPH. As expected, at low AMPH concentrations, SH-SY5Y neurons uptake DA, whereas at high AMPH concentrations, the “uptake” value is negative, indicating the release of biogenic DA. This phenomenon is shown in more detail in the time-course plot in Fig. 7b. Cells exposed to DA only (blue squares) uptake the neurotransmitter relatively slowly and plateau after 6 min of incubation (similar to the data shown in Fig. 5b), while cells exposed to DA and 10 µM AMPH (green circles) release DA quickly and plateau after less than 2 min of incubation. The amount of DA released is approximately 131 ± 16 nM (ave. ± S.D.), which is equivalent to 334 ± 51 pmol DA per mg protein. This finding is consistent with previous studies investigating dopaminergic tissues from rodent brains, in which DA bioavailability for release ranges from ~5 to 225 ng DA per mg protein (equivalent to ~30 to 1500 pmol DA per mg protein)^43,44^. Figure 7c highlights the full range of DA homeostasis (after 10 min of incubation with extracellular DA) from uptake to release.

Finally, we used the new tool to probe the mechanism of AMPH-mediated DA release. AMPH is known to (1) reverse the direction of DAT such that unpackaged intracellular DA flows into the synapse^38,45^, and has also been shown to (2) promote the release of vesicle-packaged DA^46–48^. Mechanism (1) is the canonical understanding of the effect of AMPH on DA homeostasis; mechanism (2) is somewhat controversial. We hypothesized that it could be possible to investigate the contributions of both mechanisms by pre-treating the cells with cocaine [which should inhibit mechanism (1)]^47^ prior to stimulating DA release with AMPH. Figure 7d shows two DA release curves for day-6 differentiated SH-SY5Y neurons. At time *t* = 1 min, neurons pre-incubated with 100 μM cocaine (green circles) released 76.9 ± 6.8 nM DA (ave. ± S.D.) into the extracellular space; this amount significantly differs (p = 0.0024) from that released by neurons not pre-incubated with cocaine (blue squares), which released 94.8 ± 9.7 nM. This result may imply that ~20% of biogenic DA is released by the DAT-mediated reverse transport mechanism (1), while ~80% of biogenic DA is released by mechanism (2). However, this implication is speculative as there are other potential mechanisms that might contribute to the observed results. For example, under some circumstances, AMPH has been reported to (3) deplete DA from intracellular vesicles^49^ and (4) induce DAT trafficking to the cell membrane;^50^ we propose that the rapid timing of the effects observed here makes these mechanisms less likely. In addition, under other circumstances, cocaine has been reported to enhance exocytotic DA release^51^, contributing to AMPH-mechanism (2), which might explain the slight negative DA “uptake” observed for high concentrations of cocaine in Figure 6aii. Finally, the norepinephrine transporter (NET) can also contribute to DA transport, which may further complicate the interpretation of the data shown in Fig. 7d.

Clearly, more studies are needed, and we propose that the technique described here, which is the first (to our knowledge) to be able to continuously monitor the net effect of uptake and release, could be a boon for researchers probing the complexities of neurotransmitter homeostasis.

## Conclusion

In summary, we described the only microfluidic system that we are aware of that integrates continuous real-time measurement of DA homeostasis with dopaminergic cell culture and differentiation. We propose that this uniquely flexible system, which allows for the simultaneous analysis of DA uptake and release, may facilitate interesting new experiments in the future. For example, a modified version of this system could permit real-time measurement of extracellular DA before, during, and after applying short pulses^15^ of DA-release agonists, such as AMPH, which could transiently clear stored DA to potentially generate a new model of DA-depleted Parkinsonian neurons^52^. Alternately, modified versions of this system could be amenable for evaluations of the homeostasis of other electroactive neurotransmitters, such as epinephrine^53^ and serotonin^53^. Finally, the multiplexing inherent to the system could make it useful for screening libraries of reagents to identify potential new drugs and/or therapeutic targets for neurotransmitter homeostasis.

## Materials and Methods

### Reagents

Unless otherwise specified, reagents were purchased from Sigma Chemical (Oakville, ON, Canada) or Fisher Scientific Canada (Ottawa, ON, Canada). Fluorescent dyes and cell culture reagents were purchased from Life Technologies (Burlington, ON, Canada). Antibodies were purchased from Abcam (Cambridge, UK) or Cell Signaling Technologies (CST) (Danvers, MA, USA). Prior to each experiment, fresh stock solution of DA was prepared by dissolving 1.90 mg in 10 mL of 1 × PBS buffer (pH = 7.4), resulting in a final concentration of 1.0 mM. The following two sets of serial dilutions of DA were prepared in PBS from the stock solution: (a) 1000, 500, 100, 50, 10, 5.0, and 1.0 μM, and (b) 500, 100, 50, and 10 nM. All solutions used in the DMF experiments contained a surfactant additive; when not explicitly listed, the surfactant was 0.05% Pluronic F68. Registered test kits for cocaine (C-008, 61–16), ketamine (K-002, 61–498), and AMPH (A-007, 61–05) were purchased from Sigma Chemical.

### Macroscale cell culture

SH-SY5Y cells were acquired from ATCC (Manassas, VA) and cultured in T25 flasks in a humidified incubator at 37 °C with 5% CO_2_. SH-SY5Y cells were grown in culture medium comprising 1:1 DMEM:F12 supplemented with 10% fetal bovine serum and 100 U/mL penicillin/streptomycin.

### Fabrication of DMF bottom plates

DMF bottom plates were fabricated in the cleanroom facilities of the Toronto Nanofabrication Centre (TNFC), University of Toronto. Chromium-on-glass substrates (Telic Co., Valencia, CA, USA) were patterned by photolithography and etching and then coated with ~6.5 μm Parylene-C (Specialty Coating Systems, Indianapolis, IN, USA) and ~100 nm Teflon AF (DuPont, Wilmington, DE, USA) as previously described^19^. The design features 80 chromium actuation electrodes (2 × 2 mm), connected to 8 reservoir (6 × 8 mm) and 4 waste (6 × 4 mm) electrodes. Each electrode is connected by a 150 µm-wide chromium-trace leading to a contact pad at the edge of the substrate.

### Fabrication of DMF top plates

DMF top plates were fabricated in two stages in the TNFC. During stage one, ITO coated glass substrates (Delta Technologies Ltd, Stillwater, MN) were patterned and etched as previously described^19^. Briefly, four identical ITO e-sensors were fabricated in the center of the ITO substrate surrounded by a fifth irregular ITO pattern serving as the DMF counter-electrode. Each e-sensor featured one asterisk-shaped WE with a surface area of 1.19 mm^2^ surrounded by a circular CE/RE (with an asterisk-shaped cut-out for the WE) with a surface area of 11.9 mm^2^. Each WE and CE/RE was connected by a dedicated 100 µm-wide ITO trace leading to a contact pad at the edge of the substrate. The second stage was adapted from a previously described protocol^25^ to coat DMF top plates with Teflon AF 1600 (DuPont, Mississauga, ON, Canada) punctuated with hydrophilic liftoff spots (i.e., areas with no Teflon). Briefly, the substrates from stage one (above) were cleaned, spin coated with photoresist (S1811, Dow Chemicals, Midland, Michigan, US) and exposed to UV light through a photomask. After developing, Teflon AF (1% weight/volume) was spin-coated at 3000 RPM and post baked at 175 °C for 5 min before immersion in acetone to lift-off four circular 2 mm dia. apertures through the Teflon AF. Then, the substrate was baked successively on a hot plate at 260 and 170 °C for 10 min each. After baking, each substrate had a global coating of Teflon AF with four 2 mm dia. circular (open) apertures over the e-sensors. Prior to use, each e-sensor was conditioned with poly-d-lysine and loaded with adherent SH-SY5Y cells as described in the supplementary information to form c-e-sensors.

### Two plate DMF device assembly and operation

The top and bottom plates were assembled with 150 µm thick spacers formed from double-sided tape such that the unit droplets (covering one actuation electrode) were 600 nL. Droplets were actuated by applying 95 V_RMS_ electric potentials between the DMF actuation electrodes (on the bottom plate) and DMF counter-electrode (on the top plate), which were controlled and managed using the open-source DropBot system as previously described^23^. When a unit droplet (or larger) was moved across a hydrophilic c-e-sensor, an ~470 nL sub-droplet was formed on the c-e-sensor by passive dispensing^25^. In the droplet manipulation procedures, the device was oriented with the top plate on “top,” but at all other times, the device was inverted with the top plate on the “bottom.”

### Measurement impact on cell health

SH-SY5Y cells were seeded, grown, and differentiated in 7 μL aliquots of differentiation medium for 0–6 days on c-e-sensors on DMF top plates in microincubators as described in the supplementary information (Fig. S1a-c). For the cells subjected to the “ + measurement,” every 24 h post seeding, the microincubators were removed from the culture incubator, and 5 sets of DPV measurements were collected (in series) using each c-e-sensor (see “Electrochemistry analysis” section below for details). For top plates bearing cells subjected to the “− measurement,” the microincubators were removed from the culture incubator, but no DPV measurements were collected. After “ + measurement” or “− measurement,” most microincubators were returned to the culture incubator for continued growth and analysis. After each day of differentiation, 3 microincubators from both the + /− measurement sets were sacrificed (and not cultured further). These cells were transitioned from the microincubator to a DMF device (Fig. S1d) and evaluated by confocal fluorescent imaging after staining for βIII-tubulin (see “Immunocytochemistry on DMF” section below for details). Under each such condition (+/− measurement), one image was collected from each hydrophilic spot on all three top plates (for a total of 12 images per condition) and at least 10 images were evaluated to determine the neurite length as previously described^19^ using the NeuroJ plugin for ImageJ^54^. Briefly, in each image, 15 cells were randomly selected for measurement and the length of the longest neurite on each selected cell was measured from the base of the axon hillock to the tip of the growth cone.

### Cell impact on measurement

The effect of SH-SY5Y cells occluding the working electrode on the measured DPV signal was determined in two steps. First, the cells were seeded, grown, and differentiated in 7 μL aliquots of differentiation medium for 6 days on c-e-sensors on DMF top plates in microincubators as described in the supplementary information (Fig. S1a-c). The cells were transitioned to a DMF device (Fig. S1d), and the spent differentiation medium was replaced by driving two consecutive unit-droplets of 1 µM DA in 1× PBS (pre-warmed to 37 °C) across the c-e-sensor in series, with excess going to waste. Five DPV measurements were immediately collected using each c-e-sensor (see “Electrochemistry analysis” section below for details). As a control, droplets of 1 μM DA in PBS were also evaluated under the same conditions on devices not containing any cells. Second, the cells were evaluated by confocal fluorescent imaging after staining for βIII-tubulin (see “Immunocytochemistry on DMF” section below for details), and one image was obtained per c-e-sensor.

The images obtained in step two were used to calculate the fraction of the WE surface area occluded by adherent cells as follows. (A) A mask of the working electrode was superimposed over the fluorescent image in Affinity Designer (Serif, Nottingham, England) such that only cells and parts thereof covering the electrode remained in the image; then, the image was flattened and exported as a 24-bit png file. (B) The png file was opened in ImageJ and converted to black and white using “Image:Color:RGB to Luminance.” (C) The black and white image was thresholded to create a binary image of the cell-covered area of the WE using “Image:Adjust:Threshold” with settings (min, max) = (20, 255). In this image, the pixels without cells are black, and the pixels with cells (or parts thereof) are white. (D) The number of white pixels was summed using “Analyze:Analyze Particles” with settings (size, circularity) = (1-infinity, 0–1). This value was divided by the precomputed number of pixels of the entire WE to yield the fractional coverage. Each electrochemical signal measured in step one was binned according to the fractional coverage determined in step two as follows: control (no cells), 0–3% cell coverage, 3–6% cell coverage, 6–9% cell coverage, and 9+% cell coverage. Statistical significance between bins was evaluated by one-way ANOVA.

### DA uptake assays

SH-SY5Y cells were seeded, grown, and differentiated in 7 μL aliquots of differentiation medium for 6 days on c-e-sensors on DMF top plates in microincubators as described in the supplementary information (Fig. S1a-c). The cells were transitioned to a DMF device (Fig. S1d), and the spent differentiation medium was replaced by driving two consecutive unit-droplets of 0.015, 0.03125, 0.0625, 0.125, 0.25, 0.5, or 1 µM DA in 1× PBS (pre-warmed to 37 °C) across the c-e-sensor in series, with excess going to waste. In some experiments, the 1 µM DA-containing droplets were supplemented with 0.1, 0.5, 1.0, 5.0, 10, 50, or 100 µM cocaine; 0.1, 0.5, 1.0, 5.0, 10, 50, or 100 µM EGCG; 5.0, 10, 50, 100, 500, 1000 or 5000 µM ketamine; or 0.01, 0.05, 0.1, 0.5, 1.0, 5.0, or 10 µM AMPH. Each condition was repeated on at least three devices with 4 c-e-sensors per plate for a total of 12 experiments. In all cases, the DA-containing droplets (with or without DAT ant/agonists) were incubated with the cells for 10 min, during which the DPV measurements were performed (see below).

A variation of the procedure described above was applied as a DAT pre-inhibition assay. Briefly, after the transfer of day-6 differentiated SH-SY5Y neurons to a DMF device (as above), the spent media was replaced by driving two consecutive unit-droplets of 1 × PBS (pre-warmed to 37 °C) either with (“ + inhibition”) or without (“- inhibition”) 100 μM cocaine across the c-e-sensor in series, with excess going to waste. After incubating for 10 min, the + /– inhibition solution was replaced by driving two unit-droplets containing 5 µM AMPH and 1.0 µM DA in 1× PBS across the c-e-sensor and incubated for an additional 10 min, during which the DPV measurements were performed (see below).

In most experiments, during the 10-min incubation, six DPV measurements were performed from each c-e-sensor every 2 min, beginning at 0 min (immediately after media replacement), and then after 2.0, 4.0, 6.0, 8.0, and 10 min (see “Electrochemistry analysis” section below for details). In other experiments, the DPV measurements were performed at 0, 0.5, 1.0, 2.0, and 4.0 min.

### Electrochemical analysis

The DPV measurements were performed using a DStat potentiostat (built in-house and operated as previously described^22^). Each voltammogram was acquired using a two-electrode c-e-sensor (WE and CE/RE) patterned on a DMF top-plate at 50 mV s^−1^ while scanning from 0 V to + 0.9 V, with step potential 30 mV, pulse amplitude 100 mV, pulse period 0.6 s, pulse width 50 ms, and sample period 5 ms. For each measurement, 5–10 consecutive scans were obtained, and the signal at 500 mV of the final 5 scans (for tests with 10 scans) or final 3 scans (for tests with 5 scans) was collected, averaged, and recorded.

Most experiments were conducted in 470 nL VMs generated by passive dispensing in DMF devices. In some such experiments, the VMs contained no cells and were used to interrogate the DA standards (10 nM to 1.0 µM in PBS) to generate a calibration curve. The signals measured in these experiments were plotted as a function of concentration and fit by least-squares regression. The limit of detection was defined as the concentration (from the regression) corresponding to the average signal of the blank (measured in PBS with no DA) plus three standard deviations of the signal of the blank. Other such experiments were conducted in VMs containing cells and 1.0 µM DA in PBS with and without DAT ant/agonists. The signals measured in these experiments (after various incubation times) were converted to extracellular DA concentrations (i.e., [DA]_0-min_, [DA]_2-min_, [DA]_6-min_, etc.) according to the calibration curve. DA uptake at the X-min time point was simply defined as [DA]_0-min_ − [DA]_X-min_. The DA uptake values were typically expressed in units of concentration, but in some cases were referenced to cell number and/or total cellular protein (see supplementary information for details).

Most DA uptake/release data were fit with a generic sigmoidal function. For the experiments involving the DA uptake antagonists, an IC_50_ (defined as the concentration of inhibitor at which the measured DA uptake was 50% between the minimum and maximum values of the sigmoidal fit) was generated for each data-set (12 replicates). A Dixon’s *Q* test at 95% confidence level was applied to remove outliers, and the remaining measures were averaged to calculate the reported value (and S.D.). The DA uptake kinetics experiment data were fit with the Hill equation^55^ (Eq. 1):

1 $$y = V_{{\rm{max}}}\frac{{x^n}}{{K_h^n + x^n}},$$

where *y* is the amount of DA uptake after 10-min incubation, *V*_max_ is the maximal uptake defined as the asymptote of the Hill fit, *x* is the initial concentration of DA in the droplet, *K*_h_ is the initial concentration of DA in the droplet required for half maximal uptake, and *n* is the Hill coefficient, which describes the cooperativity of the DA/DAT interaction. Each replicate dataset was plotted and fitted separately to obtain unique Hill fit parameters (*V*_max_, *K*_h_, and *n*), which were averaged to calculate the reported value (and S.D.). Finally, in still other experiments, measurements were obtained from cells in 7 μL aliquots of cell culture/differentiation medium in microincubators without any DA present.

### Immunocytochemistry on DMF

SH-SY5Y cells grown on c-e-sensors on DMF top plates were evaluated by a technique known as digital microfluidic immunocytochemistry in single cells^15^ in a 10-step procedure. Briefly, (1) the cells were fixed by dispensing and driving a unit-droplet of 4% paraformaldehyde in PBS with 0.05% Pluronic F-68 across the hydrophilic site. (2) The cells were washed by dispensing and driving two unit-droplets of PBS containing 0.05% Brij-35 across the site (in series) and (3) permeabilized by dispensing and driving a unit-droplet of 0.2% Tween 20 in PBS across the site. (4) Step two was repeated. (5) Primary antibodies were delivered to the fixed, permeabilized cells by dispensing and driving a unit-droplet containing rabbit anti-βIII-tubulin (ab18207 diluted 1:800) in 1% non-fat dry milk in PBS supplemented with 0.05% Brij-35 across the hydrophilic site. (6) Step two was repeated again, and (7) step five was repeated with secondary antibody Alexa Fluor 555 conjugated anti-rabbit (CST#4413 diluted 1:1200). (8) The top plate was detached from the DMF device and immersed in 0.1% PBS with 0.05% Tween 20, followed by (9) washing with distilled water and subsequently (10) the substrate, was allowed to air dry. The dried substrate was evaluated under an Olympus (Tokyo, Japan) IX71 microscope at ×20 or ×40 magnification with a BH2-RFL-T3 fluorescent source. When images of the entire c-e-sensor were required, sub-images were stitched together using Affinity Designer.

## Supplementary information

Supplemental Information
