# Correction to: A microfluidic platform for continuous monitoring of dopamine homeostasis in dopaminergic cells

**DOI:** 10.1038/s41378-019-0058-1

**Published:** 2019-04-22

**Authors:** Yue Yu, Richard P. S. de Campos, Seolim Hong, Dimitar L. Krastev, Siddharth Sadanand, Yen Leung, Aaron R. Wheeler

**Affiliations:** 1 Institute for Biomaterials and Biomedical Engineering, University of Toronto, 164 College St, Toronto, ON M5s 3G9 Canada; 2 Donnelly Centre for Cellular and Biomolecular Research, 160 College St., Toronto, ON M5S 3E1 Canada; 3 Department of Chemistry, University of Toronto, 80 St George St., Toronto, ON M5S 3H6 Canada; 4 Department of Human Biology, University of Toronto, 300 Huron Street, Toronto, ON M5S 3J6 Canada

**Correction to:Microsystems & Nanoengineering** (2019)5:10

10.1038/s41378-019-0049-2

published online: 11 March 2019

The legend in Fig. 4a in the previously published version of this article contained erroneous units. The correct units are given in the caption–that is, 1 µM (black), 500 nM (red), 100 nM (blue), 50 nM (cyan), 10 nM (pink), and 0 nM (1× PBS solution, brown).

