# Supplementary material for: A microfluidic platform for continuous monitoring of dopamine homeostasis in dopaminergic cells
Source: Microsyst Nanoeng. 2019 Mar 11;5:10. doi: 10.1038/s41378-019-0049-2 (PMC6409360; doi:10.1038/s41378-019-0049-2)
Supplement: Supplementary file 1 — Supplemental Information [file 41378_2019_49_MOESM1_ESM.docx]

**Supplementary Information for**

A Microfluidic Platform for Continuous Monitoring of Dopamine Homeostasis in Dopaminergic Cells

Yue Yu,^1, 2^ Richard P.S. de Campos,^2,3^ Seolim Hong,^4^ Dimitar L. Krastev,^2^ Siddharth Sadanand,^2^ Yen Leung,^2^ Aaron R. Wheeler^1, 2, 4*^

^1^ Institute for Biomaterials and Biomedical Engineering, University of Toronto, 164 College St, Toronto, ON, M5s 3G9, Canada

^2^ Donnelly Centre for Cellular and Biomolecular Research, 160 College St., Toronto, ON, M5S 3E1, Canada

^3^ Department of Chemistry, University of Toronto, 80 St George St., Toronto, ON, M5S 3H6, Canada

^4^ Department of Human Biology, University of Toronto, 300 Huron Street, Toronto, ON, M5S 3J6, Canada­

^*^ Corresponding Author:

Email: [aaron.wheeler@utoronto.ca](mailto:aaron.wheeler@utoronto.ca)

Tel: 416-946-3864

Fax: 416-946-3865

**Microincubator Fabrication and Assembly**

Microincubators were formed to allow cells to differentiate prior to experiments using digital microfluidics. Each incubator featured a poly(dimethyl siloxane) (PDMS) gasket, formed as follows. Sylgard® 184 (Dow Chemicals, Auburn, MI, USA) was mixed at a ratio of 10:1 PDMS:crosslinker, according to manufacturer’s specifications. The mixture was stirred thoroughly, poured into an in-house fabricated aluminium mold, and placed in a dissecator under vacuum for 15 minutes for air removal. Next, the PDMS mixture was cured for 30 minutes at 50°C. After cooling, the solid PDMS gasket was detached from the mold, and immersed in 70% ethanol for 30 minutes in a level 2 biosafety cabinet before air-drying and storage in a sterile Petri-dish until use. Each gasket had a rectangular shape (25 × 75 × 2 mm) with a hollow rectangular cutout (19 × 69 × 2 mm) in the middle **(Figure S1a)**.

Micro-incubators were formed by sandwiching a PDMS gasket between a DMF top plate (fabricated as described in the main text) and a Teflon-AF-coated glass slide (coated using the same spin-coating parameters described for bottom-plates in the main text). Note that the DMF ‘top’ plate actually formed the ‘bottom’ of the micro-incubator (**Figure S1b).** Immediately prior to assembly, the top, bottom, and gasket were sterilized in 70% ethanol. Each e-sensor was then coated with poly-D-lysine (PDL). Briefly, a 5 µL aliquot of 10 μM PDL in distilled water was pipetted onto each e-sensor and incubated overnight. The top plates were then washed three times with sterile water and allowed to air dry in the biosafety cabinet for 3 h.

**Microincubator Use**

Prior to each microincubator experiment, SH-SY5Y cells were cultured as described in the main text, grown to 65% confluence, detached with 0.05% Trypsin in EDTA and spun down and resuspended in retinoic acid (RA) differentiation medium [the culture medium described in the main text supplemented with 20 µM all-trans retinoic acid and 0.05% Pluronic F68]. In most experiments, cells were suspended at a density of 100,000 cells/mL, which was empirically found to result in 217 ± 13 cells adhered per spot after differentiation (see below). But in some experiments (to evaluate the effect of cell number on signal measurement), cells were suspended instead at densities of 400,000, 1 million, or 2 million cells/mL.

To begin a microincubation, a (Teflon AF-coated) glass cover of a microincubator was removed, and four 5 µL aliquots of resuspended cells was loaded onto the four PDL-coated c-e-sensors. The glass cover was replaced, and the microincubator was stored in the macroscale cell culture incubator (operating under the conditions described in the main text) to allow cells to seed the surface of the sensors. 5 hours after seeding, the microincubator was retrieved, the glass cover was removed, an additional 2 µL of retinoic acid (RA) differentiation medium was added to each culture area, the glass cover re-attached, and the microincubator replaced into the macroscale cell culture incubator. As shown in **Figure S1c**, each 7 μL droplet formed a self-contained culture chamber, providing nutrients and other factors for cell growth and differentiation. 24 hours after seeding, the microincubator was retrieved, the Teflon-AF-coated glass cover was removed, 5 µL of spent RA differentiation medium was removed from each droplet, replaced with 5 µL of fresh RA differentiation medium, and the glass cover replaced on the PDMS gasket and microincubator replaced into the macroscale cell culture incubator. This was repeated every 24 hours for 3 days, after which the medium was switched to 12-O-tetradecanoylphorbol-13-acetate (TPA) differentiation medium (identical to RA differentiation medium but supplemented with 80 nM TPA instead of RA) for another 3 days. Any non-adherent cells were washed away during the various medium replenishment steps. Cells differentiated on c-e-sensors under these conditions were (qualitatively) indistinguishable from cells treated identically in poly-l-lysine-treated well plates.

After six days of differentiation (for most experiments), or fewer days of differentiation (in experiments evaluating the differentiation progress), the cells in the microincubator were transitioned into a DMF device for droplet manipulation and analysis as follows. The external surface of the microincubator was sterilized in 70% ethanol. The glass cover and PDMS gasket were removed by inserting a scalpel between the three components along the short edge of the microincubator (**Figure S1d – top**); these components were levered apart and removed, taking care to avoid disturbing the four 7 µL droplets. 5 µL of spent media was then removed from each c-e-sensor, leaving a 2 µL droplet covering the cells. Finally, the cell-bearing ITO top plate was inverted, aligned with a DMF bottom plate such that the c-e-sensors are aligned with the electrode array below (**Figure S1d – middle, bottom**) and affixed as described in the main text.


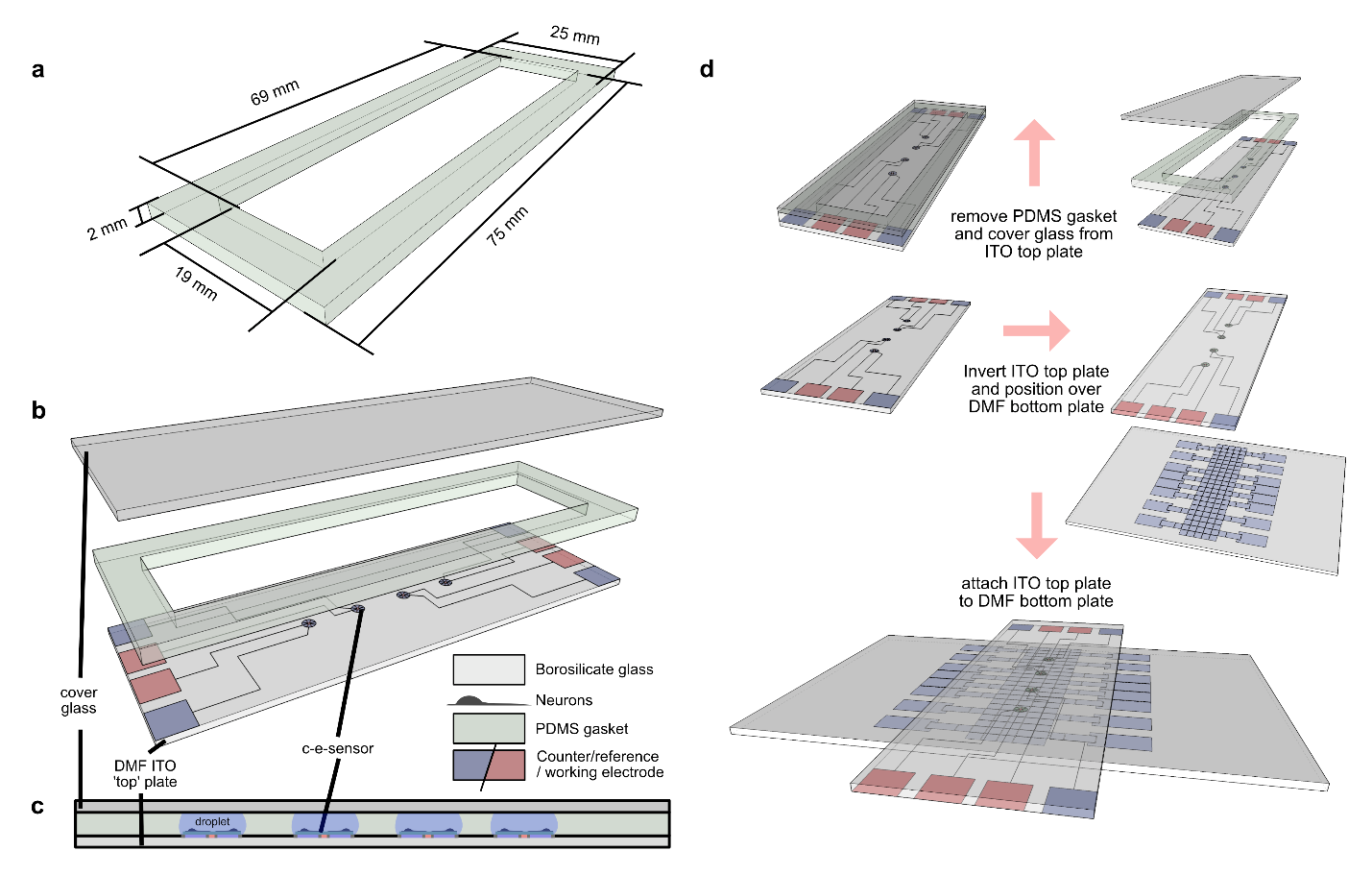


**Supplementary Figure S1** - Microincubator for multiday cell culture and differentiation. **a**Isometric computer rendering of the molded PDMS gasket used to create a top-plate microincubator. **b** Exploded-view isometric computer rendering of a microincubator, comprising a DMF ITO ‘top’ plate (light-grey, bottom) bearing four c-e-sensors (red/blue) a hollow PDMS gasket (lt. green, middle), and a Teflon-AF-coated glass cover (dark-grey, top). **c** Side-view cartoon of assembled microincubator with the DMF top-plate on the bottom, the PDMS gasket in the middle, and the glass slide on top. Each c-e-sensor bears a layer of cells growing in a 7 μL droplet (blue). **d** Series of cartoons illustrating the transition from microincubator to DMF device. First (top) the microincubator is disassembled by removing the cover glass and detaching the PDMS gasket. Second (middle) the DMF top plate is inverted and positioned above a DMF bottom plate. Third (bottom) the top and bottom plates are joined via an adhesive spacer.

**Cell Count and Protein Measurement**

In a small number of experiments, day-6 differentiated SH-SY5Y neurons seeded in microincubators at 100,000 cells/mL were used for either cell count or total protein measurement. For cell counts, three top-plates were prepared on different days, and after differentiation, 5 µL of spent media was removed from each c-e-sensor. Each remaining droplet (2 μL ea.) was supplemented with 2 µL of 1:1 Trypan Blue:1× PBS (Sigma T8154, MilliporeSigma, Oakville, Ontario, Canada) and then counted under a microscope. There were few blue (dead) cells; those that were healthy were recorded, giving a total cell count of 217 ± 13 cells/c-e-sensor (*N* = 12 c-e-sensors). For total protein measurement, three top-plates containing day-6 differentiated SH-SY5Y neurons were prepared on three different days for a total of nine top plates. Each plate was washed in a 50 mL falcon tube filled with 45 mL of 37ºC pre-warmed 1× PBS once and all liquid on each c-e-sensor replaced with a single 3 µL droplet of 0.05% Trypsin-EDTA (Thermo Fisher, Whitby, Canada) and incubated for 5 minutes. After the incubation, 7 µL of TPA differentiation medium was added to each c-e-sensor, and the entire 10 µL volume was collected by pipette for analysis. The total protein content of 184 ng/c-e-sensor or 846 ± 56 pg/cell (using the average number per c-e-sensor determined above) was determined with the Lowry method implemented according to manufacturer’s instructions (TP0300, Sigma, Oakville, Canada), measuring the absorbance at 570 nm on a Pherastar multi-well plate reader (BMG Labtech, Offenburg, Germany).

**DA Uptake/Release Conversion**

DA uptake and release concentration values converted to pmol DA per mg of cellular protein, $C_{(pmol DA/mg protein)}$, were calculated using the average DA nanomolar concentration ($X_{1}$), the volume of the virtual microwell (treated as a 470 nL constant) and the average estimates of 217 cells per c-e-sensor ($X_{2}$) and 846 pg protein per cell ($X_{3})$.

| $C_{(pmol DA/mg protein)}=\frac{X_{1}\times(470 nL)}{X_{2}\times X_{3}}$ | (S1) |
| --- | --- |

The standard deviation related to $C_{(pmol DA/mg protein)}$ was calculated based on error propagation related to unit conversions and multiplication/division operations of the measured values,

| $S.D.=C_{(pmol DA/mg protein)}\sqrt{\left( \frac{s_{1}}{X_{1}} \right)^{2}+\left( \frac{s_{2}}{X_{2}} \right)^{2}+\left( \frac{s_{3}}{X_{3}} \right)^{2}}$ | (S2) |
| --- | --- |

where $s_{1}$, $s_{2}$ and $s_{3}$ are the respective standard deviations for $X_{1}$, $X_{2}$ and $X_{3}$.
